# Comparison of two different antiseptics regarding intracutaneous microbial load after preoperative skin cleansing in total knee and hip arthroplasties

**DOI:** 10.1038/s41598-022-23070-7

**Published:** 2022-10-29

**Authors:** Sabrina Böhle, Anna-Maria Vogel, Georg Matziolis, Patrick Strube, Sebastian Rohe, Steffen Brodt, Mario Mastrocola, Henk Eijer, Jürgen Rödel, Chris Lindemann

**Affiliations:** 1 Orthopaedic Department of the Waldkliniken Eisenberg, Orthopaedic Professorship of the University Hospital Jena, Campus Eisenberg, Klosterlausnitzer Straße 81, 07607 Eisenberg, Germany; 2 Department of Orthopaedic Surgery, Spital Emmental, Oberburgstrasse 54, 3400 Burgdorf, Switzerland; 3 Institute of Medical Microbiology, Jena University Hospital, 07747 Jena, Germany

**Keywords:** Infection, Orthopaedics, Cellular microbiology

## Abstract

Periprosthetic infections (PPIs) are a serious concern in total knee and hip arthroplasty, and they have an increasing incidence. To prevent PPI, preoperative skin disinfection, as a key element of antisepsis, represents an important part of infection prevention. However, no specific antiseptic agent is endorsed by the relevant guidelines. The purpose of this retrospective, not randomized study was to investigate the difference in the residual bacteria load between an approved antiseptic with an alcohol-based solution with additional benzalkonium chloride (BAC) and an alcohol-based solution with additional octenidine dihydrochloride (OCT) at two different time periods. In 200 consecutive patients with total knee or hip arthroplasty, skin samples from the surgical sites were collected after skin disinfection with BAC (100 g solution contain: propan-2-ol 63.0 g, benzalkonium chloride 0.025 g) or OCT (100 g solution contain: octenidine dihydrochloride 0.1 g, propan-1-ol, 30.0 g, propan-2-ol 45.0 g) (100 patients per group). Following the separation of cutis and subcutis and its processing, culture was performed on different agar plates in aerobic and anaerobic environments. In the case of bacteria detection, the microbial identification was determined by matrix-assisted laser desorption ionization–time of flight mass spectrometry (MALDI-TOF MS), and the number of contaminated samples was compared between the groups. Additionally, multiple regression analysis was performed to examine the effect of the type of disinfectant, BMI, age, sex, rheumatoid arthritis, diabetes mellitus, skin disorders, smoking status, and localization of skin samples on positive bacteria detection. A total of 34 samples were positive for bacteria in the BAC group, while only 17 samples were positive in the OCT group (*p* = 0.005). Disinfectant type was the only significant parameter in the multiple regression analysis (*p* = 0.006). A significantly higher contamination rate of the subcutis was shown in the BAC group compared to the OCT group (19 vs. 9, *p* = 0,003). After the change from BAC to OCT in preoperative skin cleansing in the hip and knee areas, the number of positive cultures decreased by 50%, which might have been caused by a higher microbicidal activity of OCT. Therefore, the use of OCT in preoperative cleansing may reduce the risk of PPI in hip and knee surgery. Randomized controlled trials are required to confirm the effect and to evaluate if it reduces the risk of PPI.

## Introduction

Total or partial replacement of the knee and hip joints are frequently performed orthopaedic surgeries, and the incidence of such replacements is expected to increase in the next decades^1–3^. Consequently, an increase in periprosthetic infections (PPIs) is to be expected, with a current relative frequency of approximately 0.4–2% in primary total knee and total hip arthroplasties and 5.6% in revision surgeries^4,5^. These infections lead to high direct and indirect costs in the health care system^6^, and to a serious burden for affected patients, which can range from subsequent antibiotic treatments to removal of the prosthesis or amputation or disarticulation^5,7^.

PPIs are related to biofilms, which can be formed by bacteria of our skin flora^8^. This includes various bacteria, such as *Staphylococcus aureus, coagulase-negative staphylococci, streptococci*^9,10^, and *Cutibacterium acnes,* which also often cause prosthesis infections^11,12^.

To prevent such complications, preoperative skin disinfection, as a key element of antisepsis, represents an important part of infection prevention. Therefore, various antiseptics (e.g., alcohol, polyvidone-iodine and chlorhexidine gluconate (CHX), as well as combinations) have been studied extensively^13^. Furthermore, guidelines for prevention of surgical site infections (SSIs) recommend, with high-quality evidence, the use of preoperative skin preparation with an alcohol-based antiseptic agent^14,15^. However, due to a lack of conclusive randomized controlled trials (RCTs), no specific antiseptic agent is endorsed. Alcohol shows strong immediate antimicrobial activity but has no relevant residual activity on skin. Alcohol additives such as benzalkonium chloride (BAC), chlorhexidine (CHX) or octenidine dihydrochloride (OCT) exert a different amount of residual activity on skin once the alcohol’s immediate effect has worn off^16^. Therefore, various remanent agents could show better long-term effectiveness than alcoholic solutions and nonalcoholic antiseptics, e.g., in catheter-associated infections^17,18^. In addition to its long-lasting effect, OCT offers broad and fast-acting fungicidal, bactericidal, and partly virucidal effects at a 0.1% concentration. OCT can be used prophylactically or therapeutically^19^ and has a high effect on pathogenic microbiological burden in chronic wounds like venous leg ulcers^20^. Furthermore, in vitro studies have shown that the antimicrobial efficacy of OCT is approximately 3–10 times higher than that of CHX^21^. However, a direct comparison of both different alcohol-based antiseptics containing an additional antimicrobial agent regarding skin colonization after preoperative disinfection has not yet been performed.

Therefore, the aim of this study was to compare the efficacy of two commercially available alcohol-based antiseptic solutions for preoperative skin disinfection. Apart from alcohol, one solution contained OCT. Because of the abovementioned properties of OCT, the hypothesis was that preoperative skin cleansing before total hip or knee surgery with an alcohol-based antiseptic with additional OCT is more effective in reducing the human skin flora compared to preoperative skin cleansing with our former inhouse standard, an alcohol-based antiseptic with BAC.

## Methods

**Figure 1:**
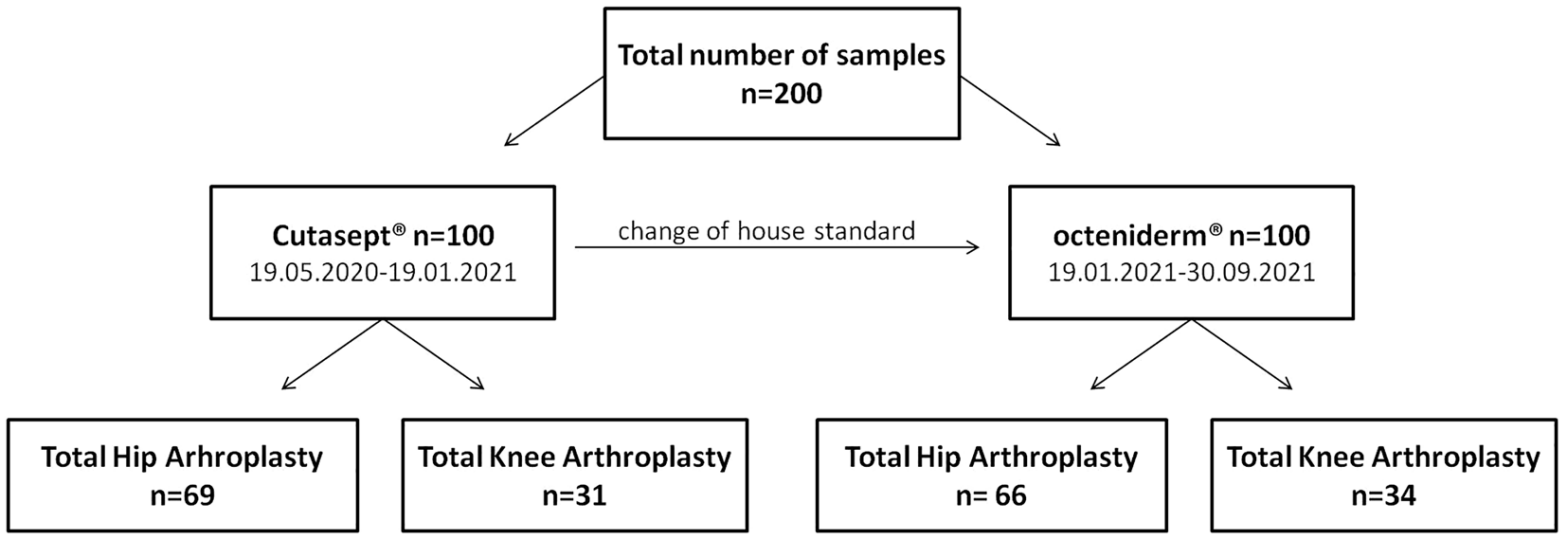
Flow chart of the skin sampling.

This was a retrospective analysis of data from a prospective, monocentriccohort study aimed at determining the residual bacterial load in the cutaneous and subcutaneous skin layers of the hip and knee after preoperative antiseptic cleansing. During the course of the prospective study, the current antiseptic solution of the preoperative cleansing standard was changed; therefore, a retrospective analysis was performed to compare the effect of the two antiseptics (Fig. 1). The study was approved by the ethics committee of the University Hospital Jena, Germany (No. 2019-1484_2-BO). All methods were carried out according to relevant guidelines and regulations based on the approval. Written informed consent for patient information and images to be published was provided by the patients or a legally authorized representative.

### Patient selection

This study included 200 patients (100 patients before and after change of the antiseptic) who were part of the described prospective study and who underwent primary implantation of knee or hip joint arthroplasty. To compare the efficacy of the different antiseptic solutions, 100 patients received preoperative skin cleansing with an alcohol-based antiseptic solution with additional BAC (BAC group), and 100 patients received an alcohol-based antiseptic solution with additional OCT (OCT group).

These patients had a sufficiently large area of skin at the surgical site that was free of irritation and ensured secure and tension-free wound closure, as assessed by the surgeon during surgery. Patients were excluded if they had scarring, skin disease, or previous surgery in the surgical area or if they had an elevated periinterventional risk profile. Factors that were considered included inadequately controlled diabetes mellitus, pathologic coagulation values, leukocytosis, CrP elevation, infections, and tumors.

The patients received detailed patient information in advance. This included a medical consultation during the inpatient admission. In addition to information about the respective operation and its risks, the original study conditions were also described, with explicit reference to additional tissue removal during the operation and possible risks. Furthermore, detailed patient information (informed consent) was distributed, which had to be signed by the informing physician and the participating patient.

### Skin removal

**Figure 2:**
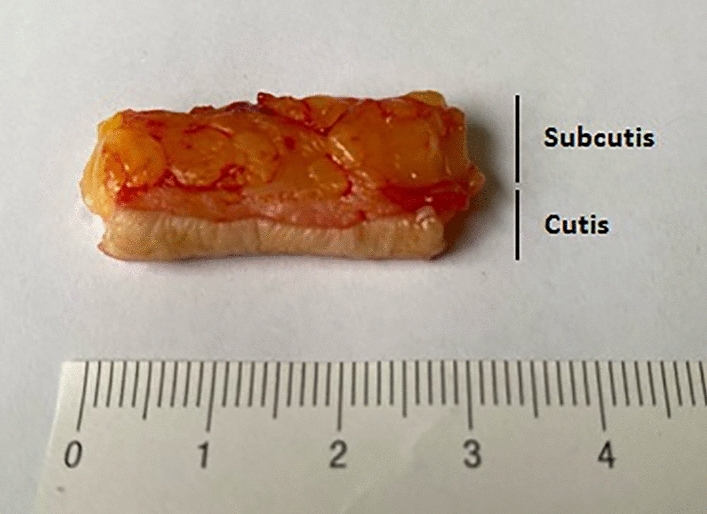
Skin samples measured approximately 3 × 0.5 × 2 cm and consisted of the skin layers cutis and subcutis.

Preoperative antiseptic disinfection of the surgical area was performed according to the current in-house standard. At the beginning of the study, this was an alcohol-based antiseptic solution (Cutasept^®^ G, Bode Chemie GmbH, Hamburg, Germany; 100 g solution contain: propan-2-ol 63.0 g, benzalkonium chloride 0.025 g) consisting of propan-2-ol with additional BAC (BAC group). During the course of the study, the entire hospital switched to an alcohol-based antiseptic solution with additional OCT (Octeniderm^®^, Shuelke & Mayer GmbH, Vienna, Austria; 100 g solution contain: octenidine dihydrochloride 0.1 g, propan-1-ol, 30.0 g, propan-2-ol 45.0 g) (OCT group). Of the 200 patients included in the study, 100 patients underwent preoperative skin cleansing with additional BAC and 100 patients underwent skin cleansing with additional OCT. A piece of tissue was removed from the patients at the beginning of their total knee or total hip endoprosthesis implantation after skin disinfection in accordance with current in-house standards and perioperative antibiotic prophylaxis (cephazolin 2 g 30 min before incision). Preoperative cleansing with four swabs for at least two minutes was performed as recommended by the manufacturer for sebaceous gland-rich skin. Tissue samples were harvested from a marginal part of the wound after skin incision so that no additional wound or scar was created. The skin samples measured approximately 3 × 0.5 × 2 cm and consisted of the skin layers cutis and subcutis (Fig. 2). Immediately after collection, the skin sample was packed dry in a sterile compress in a sterile container and stored in the refrigerator at 8 °C. The samples were in the refrigerator for an average of about 1 h, but no longer than two hours.

### Sample preparation and microbiological diagnostics

In the further sample preparation, no inactivation substances for BAC and OCT were used. In the laboratory under sterile conditions, the skin sample was first separated into two layers, cutis and subcutis, each of which was weighed to 350 mg to achieve quantitative comparability (Fig. 2). The respective samples were then packed into separate tubes and homogenized using a Precellys 24 tissue homogenizer (Bruker Instruments, Montigny-le-Bretonneux, France) with the addition of 2 ml sterile Ringer's solution (3 × 10 s with intermediate cooling on ice for two minutes each). The tissue, which had been reduced in size by the homogenizer, was then spread by pipette onto suitable culture media with 300 µl each (chocolate agar, Columbia sheep blood agar, Schaedler agar (BD Diagnostics, Heidelberg, Germany)) and incubated under aerobic and anaerobic (Schaedler agar) conditions at 37 °C for seven days. Enrichment cultures were performed by additional incubation of 20 µl of the homogenized solution on a thermoshake into a brain–heart broth (BD Diagnostics). The incubated brain–heart broth was kept continuously agitated on the thermoshake at 37 °C and checked daily for turbidity. If this was the case, we plated it out on chocolate and Columbia sheep blood agar and checked the plates incubated under aerobic conditions for visible CFUs after 48 h. Bacterial colonies grown on agar plates were identified by Vitek MS (bioMérieux, Nürtingen, Germany). The detection limit was 1 CFU/350 mg examined tissue (cutis respectively subcutis). 1 CFU was considered as a positive germ detection). If CFUs could no longer be distinguished due to excessive growth on the agar plate, this sample was considered a contaminant and was not included in the evaluation.

**Figure 3:**
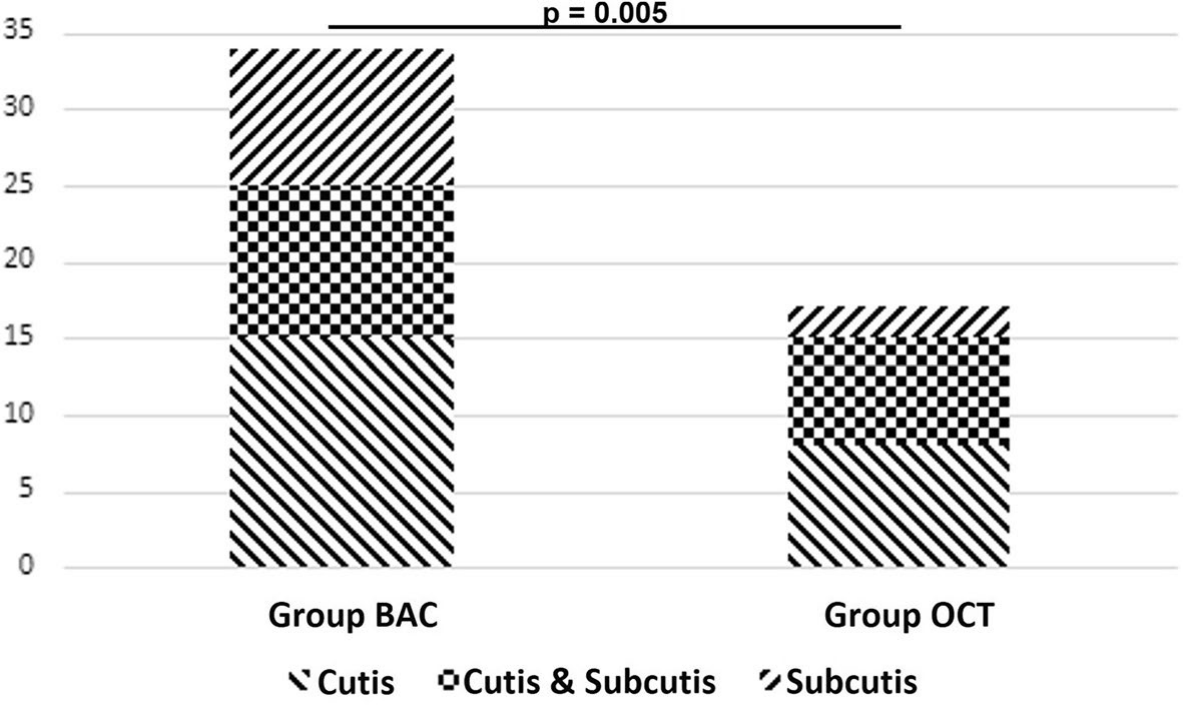
Bacteria detection in different skin layers.

### Statistical analysis

The statistical evaluation was performed using SPSS Statistics Version 24 software for Macintosh (IBM, Armonk, USA). The demographic data were assessed using Student's t test for independent samples, and the normal distribution of the data was assessed in advance using the Kolmogorov–Smirnov test. Categorical data were compared using Pearson’s X^2^ and Fisher’s exact tests, and continuous data were compared using Student's t test. Based on the primary hypothesis, a single-sided significance check was performed to examine the superiority of the OCT group compared to the BAC group in residual bacteria detection after skin cleansing. All other tests were double-sided, and a *p* value < 0.05 was considered to indicate statistical significance for all statistical tests. Multiple regression analysis was performed to examine the effect of the type of disinfectant, BMI, age, sex, rheumatoid arthritis, diabetes mellitus, skin disorders, smoking status, and localization of skin on positive bacteria detection in the skin after preoperative cleansing.

### Ethical approval and informed consent

The study was approved by the ethics committee of the University Hospital Jena, Germany (2019-1484_2-BO). Written informed consent for patient information and images to be published was provided by the patients.

## Results

### Baseline demographics

Baseline characteristics are shown in Table 1. A total of 135 patients underwent THA, and 65 patients underwent TKA. There were no statistically significant differences between the groups for any demographic or baseline characteristics. Patient-related risk factors such as BMI, age, immunosuppression, and smoking status had no influence on bacterial detection in the skin after preoperative cleansing.

**Table 1:** Baseline demographics and clinical characteristics.

Variable	Total (n = 200)	BAC group (n = 100)	OCT group (n = 100)	*P*-value
Age (years)	66.4 ± 9.8	66.7 ± 10.4	66.1 ± 9.1	0.636
Male	94	50	44	0.395
Female	106	50	56	0.395
BMI (kg/m2)	29.6 ± 5.0	29.4 ± 5.2	29.8 ± 4.7	0.592
BMI > 30 kg/m2	91	45	46	1.000
Smoker	25	13	12	1.000
Frequent alcohol consumption	109	57	52	0.570
Rheumatoid arthritis	7	5	2	0.445
Immunosupression	8	6	2	0.279
Diabetes mellitus	24	13	11	0.414
Skin disorders	1	0	1	1.000
Total hip arthroplasty	135	69	66	0.651
Total knee arthroplasty	65	31	34	0.651

BAC group: alcohol-based antiseptic with additional benzalkonium chloride; OCT group: alcohol-based antiseptic with additional octenidine dihydrocloride.

#### Bacterial detection

Regarding the positive bacterial detection of the microbiologically examined skin probes (at least one probe in cutis or subcutis positive), 34 detections in the BAC group were observed, in contrast to 17 detections in the OCT group. This difference was statistically significant (*p* = 0.005, Table 2, Fig. 3). Regarding the examined superficial skin layer, 25 patients who underwent preoperative cleansing in the BAC group showed a positive bacterial detection in the cutis (10 patients without additional bacterial detection in the subcutis and 15 patients with additional positive bacterial detection in the subcutis) versus 15 patients with positive bacterial detection in the cutis who underwent preoperative cleansing with an antiseptic with additional OCT (8 patients without additional bacterial detection in the subcutis and 7 patients with additional positive bacterial detection in the subcutis). No statistically significant difference was observed between the groups (*p* = 0.55; Tables 2 and 3). Regarding the deep skin layer, 19 patients in the BAC group showed a positive bacterial detection in the subcutis (7 patients without additional bacterial detection in the cutis and 15 patients with additional positive bacterial detection in the cutis) versus 9 patients with positive bacterial detection in the subcutis in the OCT group (7 patients without additional bacterial detection in the subcutis and 2 patients with additional positive bacterial detection in the subcutis). This difference was statistically significant (*p* = 0.033, Tables 2 and 3). Regarding the quantitative count of CFUs in the cutis, no statistically significant differences were observed between the groups (M = 2, SD = 5 in the OCT group, M = 2, SD = 6 in the BAC group; *p* = 0.365). These results could also be observed in the subcutis (M = 1, SD = 5 in the OCT group, M = 2, SD = 5 in the BAC group; *p* = 0.268).Only the type of skin disinfectant showed a significant influence on the positive bacteria detection in regression analysis (*p* = 0.006).

**Table 2:** Comparison of the number of bacteria detections in cutis and subcutis between the alcohol-based antiseptic with additional benzalkonium chloride (BAC group) and the antiseptic with additional octenidine dihydrochloride (OCT group).

Cutis	Subcutis	Total (n = 200)	BAC group (n = 100)	OCT group (n = 100)	*P*-value
At least one positive		51	34	17	0.005
Pos	Pos or neg	40	25	15	0.055
Pos or neg	Pos	28	19	9	0.033

Pos = positive bacteria detection; Neg = negative bacteria detection.

**Table 3:** Comparison of the possible bacteria detection combinations in cutis and subcutis between the alcohol-based antiseptic with additional benzalkonium chloride (BAC group) and the antiseptic with additional octenidine dihydrocloride (OCT group).

Cutis	Subcutis	Total	BAC group (n = 100)	OCT group (n = 100)	*P*-value
Pos	Neg	23	15	8	0.091
Pos	pos	17	10	7	0.307
Neg	Neg	149	66	83	0.005
Neg	Pos	11	9	2	0.029

Pos = positive bacteria detection; Neg = negative bacteria detection.

*Micrococcus luteus* was the most common bacterium in the BAC group, and *Staphylococcus epidermidis* was the most common bacterium in the OCT group (Table 4). We found *Staphylococcus epidermidis* (BAC group 4%, OCT group 5%), *Staphylococcus capiti*s (BAC group 3%, OCT group 2%), *Staphylococcus hominis* (BAC group 0%, OCT group 3%), *Staphylococcus haemolyticus* (OCT group 2%), *Staphylococcus lugdunensis* (BAC group 3%), *Staphylococcus aureus* (OCT group 1%), *Micrococcus luteus* (BAC group 9%, OCT group 2%) and *Bacillus spp.* (BAC group 6%, OCT group 2%). A significantly higher concentration of polymicrobial bacteria was observed in the OCT group than in the BAC group (14 vs. 3, *p* = 0.005, Table 4).

**Table 4:** Bacterial growth after skin cleansing with the alcohol-based antiseptic with additional benzalkonium chloride (BAC group) and the antiseptic with additional octenidine dihydrocloride (OCT group).

Bacteria	BAC group (n = 100)	OCT group (n = 100)	*P*-value
*Staph. epidermidis*	4	5	n.s
*Staph. aureus*		1	n.s
*Staph. capitis*	3	2	n.s
*Staph. haemolyticus*		2	n.s
*Staph. lugdunensis*	3		n.s
*Staph. hominis*		3	n.s
*Staph.saccharolyticus*		2	n.s
*Paracoccus yeei*	2		n.s
*Phyllobacterium myrsinacearum*	2		n.s
*Micrococcus luteus*	9	2	n.s
*Bacillus spp.*	6	2	n.s
*Bacillus spc*	4		n.s
*Bacillus megaterium*	1		n.s
*Bacillus simplex*	1	1	n.s
Polymicrobial, n*	14	3	0.005

*More than one different bacterial species grown from cutis and subcutis.

N.s. = not significant.

## Discussion

The aim of the study was to compare the efficacy of two commercially available, alcohol-based antiseptic solutions for preoperative skin disinfection. We confirmed the hypothesis that preoperative skin cleansing before total hip or knee surgery with an alcohol-based antiseptic with additional OCT is more effective in reducing the human skin flora than an alcohol-based antiseptic with BAC.

Therefore, OCT lowered the intracutaneous bacteria load and led to a reduction in polymicrobial bacteria detection compared to an antiseptic with BAC. To the best of our knowledge, this is the first study investigating the effectiveness of OCT-containing solutions in comparison to BAC-containing solutions. Thus, apart from the alcohol component, OCT appears to have an additional antimicrobial effect. OCT readily binds to negatively charged surfaces, such as microbial cell envelopes, eukaryotic cell membranes, and lipid-containing bacterial cell membrane components, resulting in high antimicrobial activity^22^. Since OCT binds readily to negatively charged surfaces and is not absorbed percutaneously, at least part of the applied substance remains at the application site, thus exerting a sustained antimicrobial effect (remanent effect)^22,23^. Even with chronic wounds OCT could be a prevention of infection^24^ because of a high effect on pathogenic microbiological burden^20^. Moreover, the antimicrobial effect of cell-bound OCT is far superior to that of chlorhexidine^23^. In vitro studies showed the high antimicrobial effectiveness of OCT, which was 3–10 times higher than that of chlorhexidine^25^. In addition to the effectiveness of an antiseptic solution, the safety of the antiseptic for clinical use is important: octenidine is virtually not absorbed through the skin, and toxic side effects or systemic interactions, when OCT is used on intact skin, are not to be expected^22^.

The main source of PPIs is the patient’s own normal skin flora^9,10^. According to previous studies on intraoperative contamination, surgical site infection and deep infections, staphylococci were the most isolated organisms^26–28^. In contrast to the results of the current study, Dörfel et al*.* showed that in the shoulder after cleansing with povidone-iodine alcohol or chlorhexidine-alcohol, the majority of the bacteria on anaerobic plates were *Cutibacterium acnes,* while the majority of the bacteria on aerobic plates was *coagulase-negative staphylococci* (mainly *S. epidermidis, S. hominis, S. saprophyticus* and *S. lugdunensis*), which accounted for more than 70%. *S. aureus and M. luteus* accounted 6% of aerobic flora^29^. The difference can be explained by the different sampling locations. In the shoulder area, *C. acnes* infections are predominant^30^. The main reservoir of *C. acnes* is located deep in the skin (hair follicles and pilo-sebaceous glands)^31,32^. This might explain the different results of this study.

The data of Maurer et al*.*^33^ were also different: they found in the hip that only 12 of 60 (20%) patients were colonized with *C. avidum,* while most of the patients were colonized with other bacteria, such as coagulase-negative staphylococci (47, 78.3%), *C. acnes* (11, 18.3%), *Corynebacterium* sp. (4, 6.7%), *Cutibacterium granulosum* (2, 3.3%), *Enterococci* sp*.* (2, 3.3%), *S. aureus* (2, 3.3%).

We were able to detect a total of 34 positive bacteria in the BAC group and 25 positive bacteria in the OCT group from each 100 samples of the hip and knee. In contrast, Maurer et al*.*^33^ was able to detect bacteria in 51 of 60 samples (85%) in the hip. Regarding only the hip, we found 27 positive samples in the BAC group (39,1%) and 11 positive samples in the OCT group (16,7%). A reason for this may be that in the abovementioned study by Maurer et al*.* disinfection was performed with povidone-iodine/alcohol. However, they demonstrated that standard skin antisepsis with povidone-iodine/alcohol combined with antibiotic prophylaxis incompletely removed *Cutibacterium avidum* from the groin at the time of surgical skin incision. Factors such as the number of cultures taken, the timing, the site of sampling and the sensitivity of culture methods might also contribute to the variation in the reported contamination.

Mastrocola et al*.*^34^ confirmed in a meta-analysis of 8 studies that surgical skin preparation with chlorhexidine alcohol is more effective than povidone-iodine in reducing bacterial counts on human skin. In contrast, according to Dörfel et al*.*^29^ povidone-iodine alcohol either better reduces aerobic flora or more significantly anaerobic flora of the shoulder after surgical skin preparation compared to chlorhexidine alcohol. Here, in contrast to our method, only the superficial skin layer was tested using a standardized cup-scrub technique^35^. Numerically, as expected, there were more detections of bacteria in the cutis than in the subcutis. Nevertheless, our study showed a significant difference in the detection of germs in the subcutis between OCT and BAC. A possible explanation for this finding is the continued activity of the different antiseptics used after skin separation, since no inactivating substances were used after skin removal.

Another difference between clinical studies is seen in the perioperative prophylaxis with antibiotics: in the present study (patients with arthroplasty), we routinely used perioperative antibiotics, whereas Dörfel et al*.*^29^ did not report such a procedure (patients with shoulder surgery). Mastrocola et al*.*^34,36^ concluded that surgical skin preparation should be optimized to minimize normal skin flora, but other strategies, such as shortening the duration of surgery and the type of systemic antibiotic prophylaxis, must be considered to reduce the rate of surgical site infections.

### Limitations

Our study is not without limitations, including its retrospective design concerning the comparison of the two antiseptics. First, the study was not blinded or randomized. Second, possible contamination during processing of the sample cannot be ruled out despite sterile procedures. Third, the bacteria load of the skin was used as an end point to compare the effectiveness of the two antiseptics instead of periprosthetic infections. Further prospective follow-up studies comparing the two solutions should focus on the clinical end point “infection” and could compare the bacterial detection of the infection with the detected bacteria in this study. From this, further preoperative prophylaxis and additional knowledge may be derived. Moreover, another limitation is that the antiseptics tested were not used in the same period. However, the surgery was performed in the same manner, there was no change in personnel or change in perioperative standards. Last, we could not draw any conclusion about the residual effect of the two antiseptics since tissue samples were taken only at one timepoint. However, the residual effect after initial disinfection was not the focus of this study. Furthermore, skin removal after more than 2 h is not applicable since the average duration of hip and knee replacement is less than 1–1.5 h.

## Conclusion

Our study demonstrates that preoperative skin cleansing is not efficient in eliminating all colonizing bacteria. However, an antiseptic based on an alcohol-based solution with additional OCT significantly lowers the cutaneous bacteria load compared to BAC in hip and knee replacement surgeries. For the prevention of periprosthetic infections, the use of antiseptics with additional OCT should be further evaluated in randomized clinical trials. Furthermore, it would be interesting to find out whether there is a difference in the incidence of postoperative wound infections between the two groups.

## Data Availability

The datasets generated and analysed during the current study are not publicly available due data privacy reasons but are available from the corresponding author on reasonable request.
